# Tanshinone IIA sulfonate protects against cigarette smoke-induced COPD and down-regulation of CFTR in mice

**DOI:** 10.1038/s41598-017-18745-5

**Published:** 2018-01-10

**Authors:** Defu Li, Jian Wang, Dejun Sun, Xuefang Gong, Hua Jiang, Jiaze Shu, Ziyi Wang, Zhen Long, Yiguan Chen, Zili Zhang, Liang Yuan, Ruijuan Guan, Xue Liang, Ziying Li, Hongwei Yao, Nanshan Zhong, Wenju Lu

**Affiliations:** 1 State Key Laboratory of Respiratory Diseases, Guangzhou Institute of Respiratory Disease, The First Affiliated Hospital, Guangzhou Medical University, Guangzhou, Guangdong, China; 2 Department of Laboratory Medicine, The First Affiliated Hospital, Guangzhou Medical University, Guangzhou, Guangdong, China; 3 Sino-French Hoffmann Immunology Institute, Guangzhou Medical University, Guangzhou, Guangdong, China; 4 Department of Respiratory Medicine, The People’s Hospital of Inner Mogolia, Hohhot, 010020 China; 5 Faculty of Biology, Medicine and Health, University of Manchester, Manchester City, Britain, UK

## Abstract

Chronic obstructive pulmonary disease (COPD) is a chronic lung disease characterized by abnormal inflammation, persistent and progressive lung function decline. The anti-inflammatory actions of tanshinone IIA, which is the most important active component from Chinese herbal medicine Danshen, have been well studied. However, it remains unknown whether sodium tanshinone IIA sulfonate (STS) protects against the development of COPD. Here we found that STS inhalation (5 mg/kg, 30 min per session, twice a day) significantly attenuated lung function decline, airspace enlargement, mucus production, bronchial collagen deposition, inflammatory responses and oxidative stress caused by cigarette smoke (CS) and lipopolysaccharide (LPS) exposures in mice. Moreover, treatment with STS (10 μg/ml) reduced CS extract (CSE)-induced IL-6 and IL-8 secretion in human bronchial epithelial (16HBE) cells. The anti-inflammatory actions of STS were associated with inhibition of ERK1/2 and NF-κB activations. Interestingly, STS inhibited CS-induced reduction of cystic fibrosis transmembrane conductance regulator (CFTR) in mouse lungs and in 16HBE cells. Treatment with a specific CFTR inhibitor CFTR-Inh172 augmented CSE-induced ERK1/2 and NF-κB-dependent inflammatory responses, but abolished the inhibitory action of STS on IL-6 and IL-8 secretion in 16HBE cells. These results demonstrate that CS-induced COPD and down-regulation of CFTR are prevented by STS.

## Introduction

Chronic obstructive pulmonary disease (COPD), currently the fourth predominant cause of death in the world, is predicted to be the third one by 2030^1^. COPD is a common preventable and treatable disease featuring progressive and not fully reversible airflow limitation, which is associated with chronic inflammatory responses to poisonous particles or gases in the airway and lung^2^. The well-defined risk factor of COPD is tobacco smoking, including second-hand smoke exposure^3^. A strong relationship is identified among sustained tobacco use, oxidative stress, and COPD exacerbation in COPD subjects^4^. Although COPD is thought to be a treatable disease, existing treatment can only slow down the progression of this disease. Various treatments help to improve lung functions, reduce exacerbation and increase life quality of COPD patients, but bring huge financial burden in return^5^. At present, the strategy for COPD treatment focuses on down-regulating airway smooth-muscle tone using bronchodilators and reducing pulmonary inflammation using inhaled corticosteroids or the phosphodiesterase type 4 (PDE4) inhibitors (e.g., roflumilast)^6^. However, cigarette smoke (CS) exposure is linked to glucocorticoid resistance^7^. The broad spectrum of anti-inflammatory drugs like PDE4 inhibitors are expensive and have side effects, which limits their clinical use^8^. Therefore, it is necessary to develop new drugs targeted at immune homeostasis, redox balance as well as slowing down COPD progression with high efficiency and low side effects.

Sodium tanshinone IIA sulfonate (STS), a water-soluble substance, derives from tanshinone IIA (TIIA). TIIA is the main pharmacological component from Chinese herbal medicine Danshen, the dried root of Saviamiltiorrhiza. Danshen has been widely used in many countries including China, Japan and the United States to treat cardiovascular and cerebrovascular diseases with few adverse effects^9^. In recent years, STS and TIIA have been found to be anti-inflammatory in experimental lung and cardiac diseases^10–12^. In these experiments, STS or TIIA was administered via intraperitoneal injection. However, intraperitoneal injection may bring poor treatment compliance. Furthermore, excess STS will be administered intraperitoneally so as to reach effective concentration in lungs, which inevitably limits its clinical use. More importantly, it is not known whether STS attenuates CS-induced inflammatory responses and subsequent COPD.

In this study, we evaluated effects of STS inhalation on the development of COPD in mice exposed to CS. We also investigated the mechanisms underlying the protective roles of STS on lung inflammatory and injurious responses to CS *in vivo* mouse lungs and *in vitro* lung epithelial cells. To the best of our knowledge, this is the first report regarding the effects of STS inhalation on CS-induced COPD and underlying mechanisms.

## Results

### STS inhalation protects against the development of CS-induced COPD

**Figure 1:**
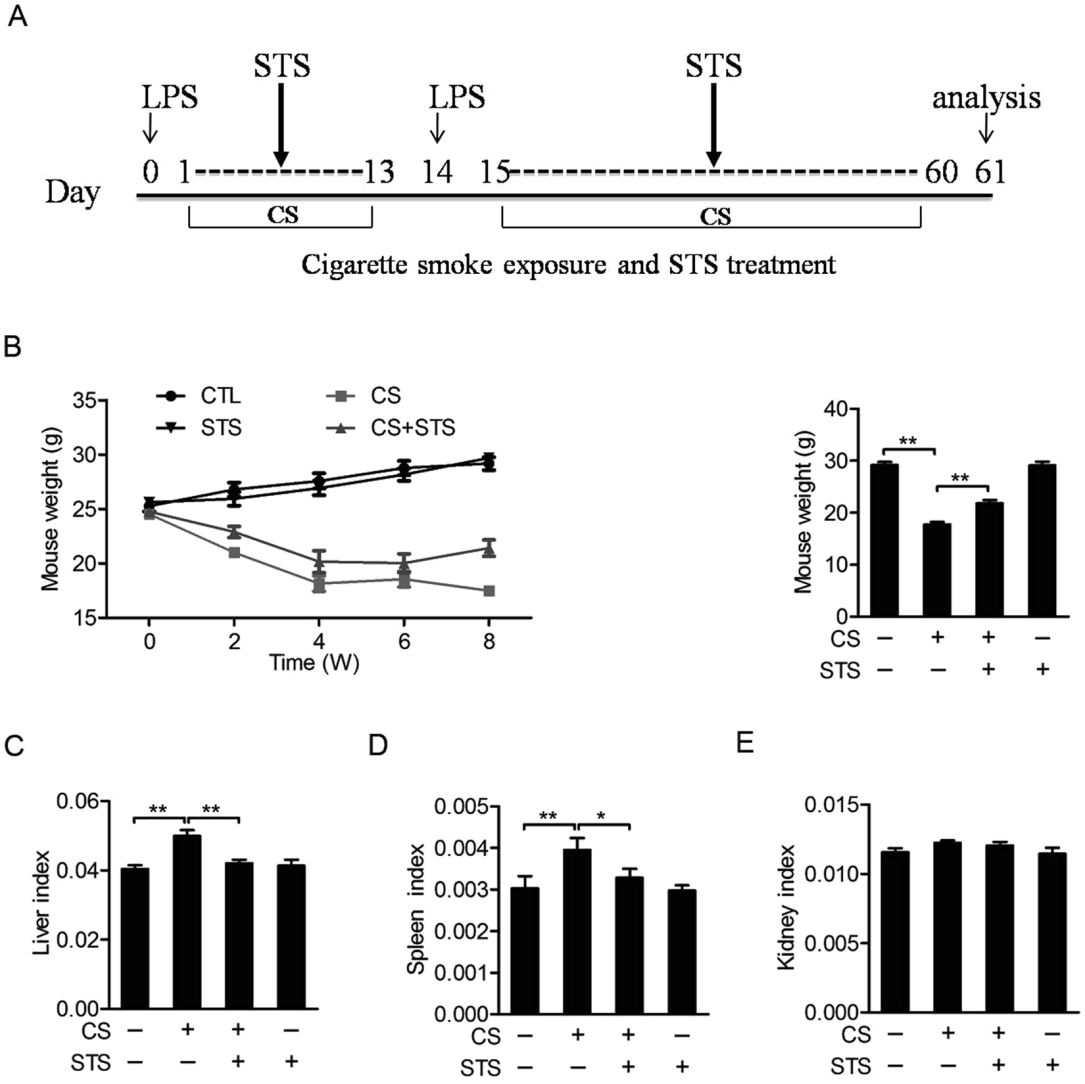
STS attenuates CS-induced body weight loss, increases of liver and spleen indices in mice. Mice with LPS intranasally inhaled (7.5 μg/mouse in 50 μl saline) at the 1^st^ and 14^th^ day were exposed to CS from day 0 to 60 except for the days giving LPS. Aerosol STS (5 mg/kg) was given to mice 30 min before CS exposure. (**A**) The scheme of CS plus LPS expousures as well as STS treatment. (**B**) During the experiments, mouse weight was monitored. At the end of the experiment, (**C**) the weight ratios of liver to whole body, (**D**) spleen to whole body and (**E**) kidney to whole body were measured. *P < 0.05 and **P < 0.01 (n = 8–11 mice per group). LPS, lipopolysaccharide; STS, Sodium tanshinone IIA sulfonate; CS, cigarette smoke.

**Figure 2:**
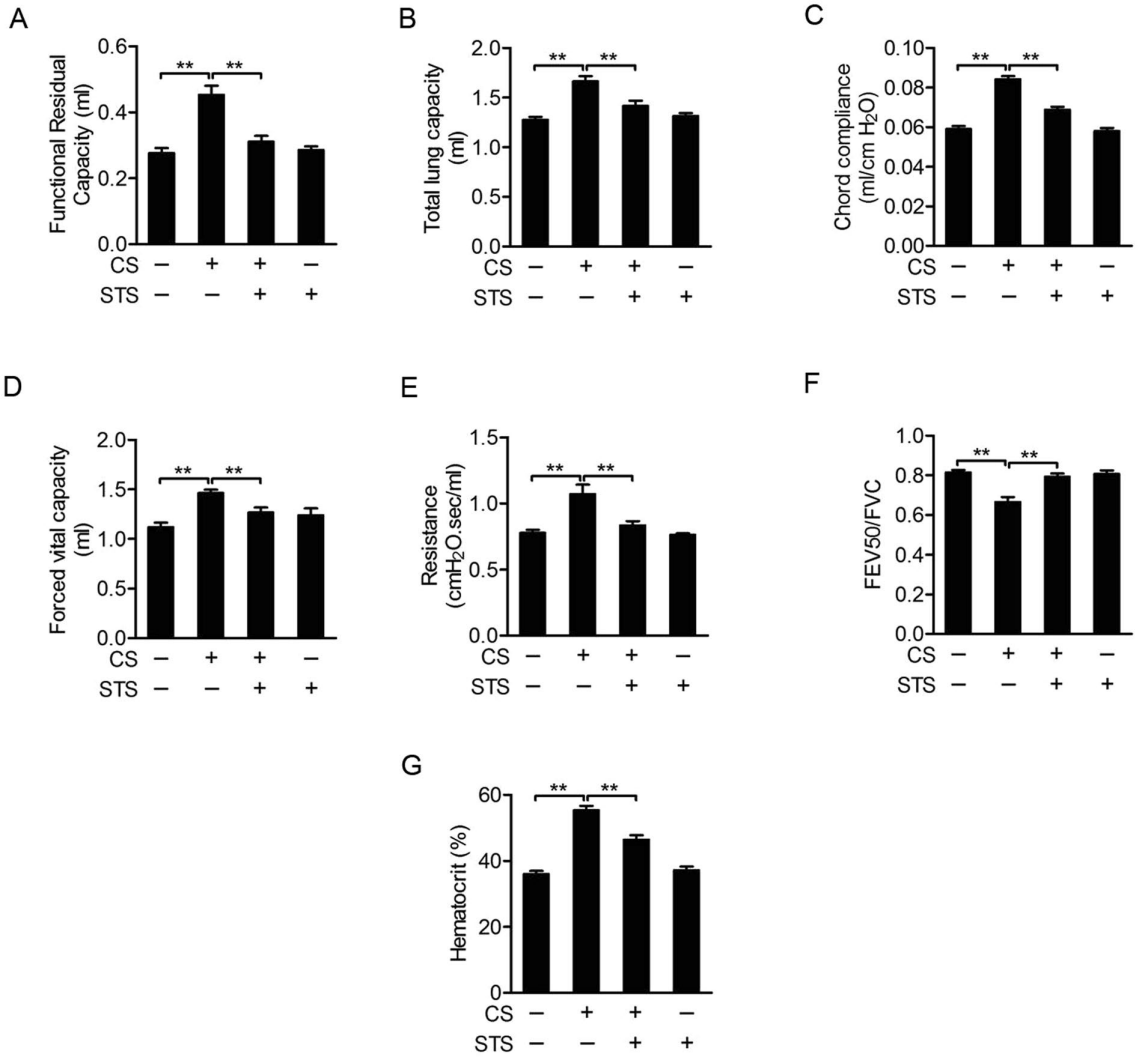
STS ameliorates CS-induced lung function decline and increase in hematocrit in mice. Lung function parameters, including functional residual capacity (**A**), total lung capacity (**B**), chord compliance (**C**), forced vital capacity (**E**), resistance index (**G**) and FEV50/FVC (**F**), were measured by the Forced Pulmonary Maneuver System. Comparison of hematocrit (**I**) among control (CTL), CS, CS plus STS (CS + STS) and STS-treated mice *P < 0.05 and **P < 0.01 (n = 8–11 mice per group). STS, Sodium tanshinone IIA sulfonate; CS, cigarette smoke.

In this model, mice given intranasal inhalation of LPS twice plus CS exposure (Fig. 1A) exhibited typical clinical manifestations of COPD. This included body weight loss (Fig. 1B) and lung function decline (Fig. 2) compared with control mice. Lung function decline was reflected by an increase in functional residual capacity (FRC) (Fig. 2A), total lung capacity (TLC) (Fig. 2B), chord compliance (Cchord) (Fig. 2C), forced vital capacity (FVC) (Fig. 2D) and lung resistance index (RI) (Fig. 2E), as well as a decrease in forced expiratory volume at 50 ms (FEV50)/FVC (Fig. 2F). All these manifestations were attenuated in mice administered with STS compared to vehicle-treated mice exposed to CS and LPS (FRC: CS group: 163.85 ± 11.08%, CS + STS group: 112.54 ± 7.06%; TLC: CS group: 130.06 ± 4.47%, CS + STS group: 110.89 ± 4.57%; Cchord: CS group: 142.6 ± 2.95%, CS + STS group: 116.54 ± 2.56%; FVC: CS group: 131 ± 3.27%, CS + STS group: 113.33 ± 5.09%; RI: CS group: 137.97 ± 9.66%, CS + STS group: 107.88 ± 4.42%; FEV50/FVC: CS group: 81.67 ± 3.33%, CS + STS group: 97.24 ± 2.05%, percentage of the CTL group, Fig. 2A–F). As shown in Fig. 2G, LPS and CS exposures significantly increased hematocrit value in blood, which was ameliorated by STS administration (CS group: 124.70 ± 2.87%, CS + STS group: 108.63 ± 2.77%, percentage of the CTL group). Furthermore, CS-induced increases of liver and spleen indices were attenuated by STS treatment (Liver index: CS group: 119.91 ± 4.07%, CS + STS group: 101.85 ± 2.44%; Spleen index: CS group: 124.70 ± 2.87%, CS + STS group: 106.63 ± 7.33%, percentage of the CTL group), while kidney index was not changed (CS group: 105.76 ± 1.58%, CS + STS group: 104.97 ± 2.24%, percentage of the CTL group, Fig. 1C–E). Altogether, these results demonstrate that STS inhalation ameliorates lung function decline and hypoxia induced by LPS and CS exposures. The dose of STS (5 mg/kg, 30 min per session, twice per day) used here to treat COPD did not cause a detectable side effect.

### STS inhalation attenuates CS-induced emphysema, collagen deposition in the small airway, goblet cell hypertrophy and hyperplasia of airway epithelium

**Figure 3:**
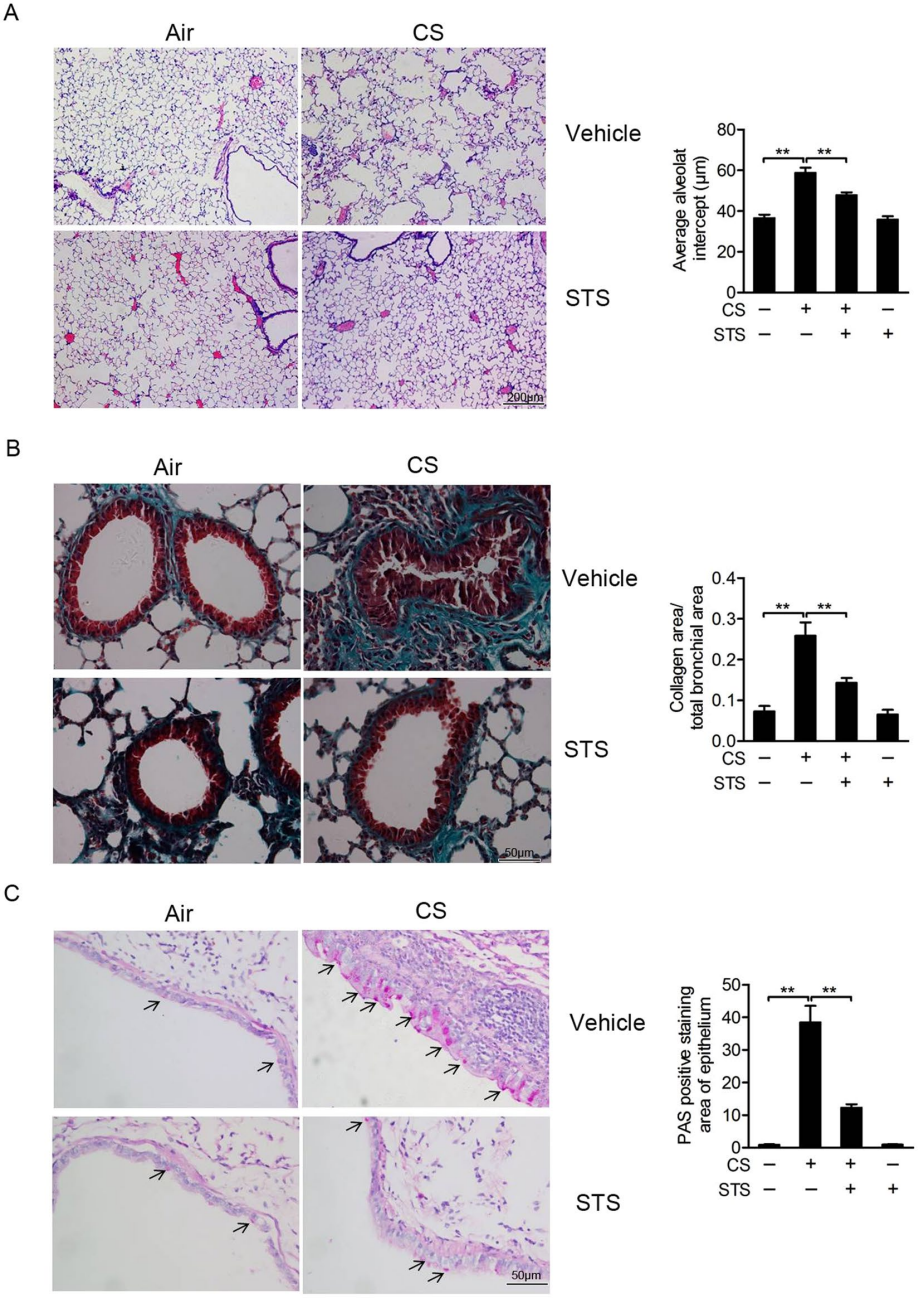
STS attenuates CS-induced emphysema, inflammation, remodeling of the airway and goblet cell hypertrophy and hyperplasia of the airway epithelium. Comparison of H&E or Masson or PAS staining of lung sections from CTL, CS, CS plus STS (CS + STS) and STS-treated mice was performed. Software IPP6.0 was used to assess the average linear intercept (Lm) of alveoli (**A**), small airway remodeling (**B**) and goblet cell hyperplasia (**C**) in at least three fields of lung section per mouse. *P < 0.05 and **P < 0.01 (n = 5 mice per group). STS, Sodium tanshinone IIA sulfonate; CS, cigarette smoke.

As shown in Fig. 3A, typical pathological manifestations of COPD, damaged alveolar walls and pulmonary bullae were found in mouse lungs exposed to CS and LPS (Average alveolar intercept: CTL group: 36.6 ± 1.62 μm, CS group: 58.8 ± 2.49 μm). Moreover, STS inhalation significantly reduced pulmonary structural damage caused by CS and LPS exposures (Average alveolar intercept: CS group: 160.65 ± 6.82%, CS + STS group: 130.61 ± 3.70%, percentage of the CTL group). Compared to the control group, exposure to CS led to significant increase in collagen deposition in the small airway, which was reduced by STS administration (Collagen area/total bronchial area: CS group: 449.83 ± 57.61%, CS + STS group: 248.76 ± 20.57%, percentage of the CTL group, Fig. 3B). Goblet cells from airway epithelium of CS-exposed mice were attenuated by STS treatment (PAS positive staining area of epithelium: CS group: 4,204.48 ± 553.89%, CS + STS group: 1,546.85 ± 211.35%, percentage of the CTL group), which was identified by PAS staining (Fig. 3C). These results suggest that STS inhalation ameliorates CS/LPS-induced pathological changes in mouse lungs.

### STS inhibits CS-mediated mucus hypersecretion in mouse airway

**Figure 4:**
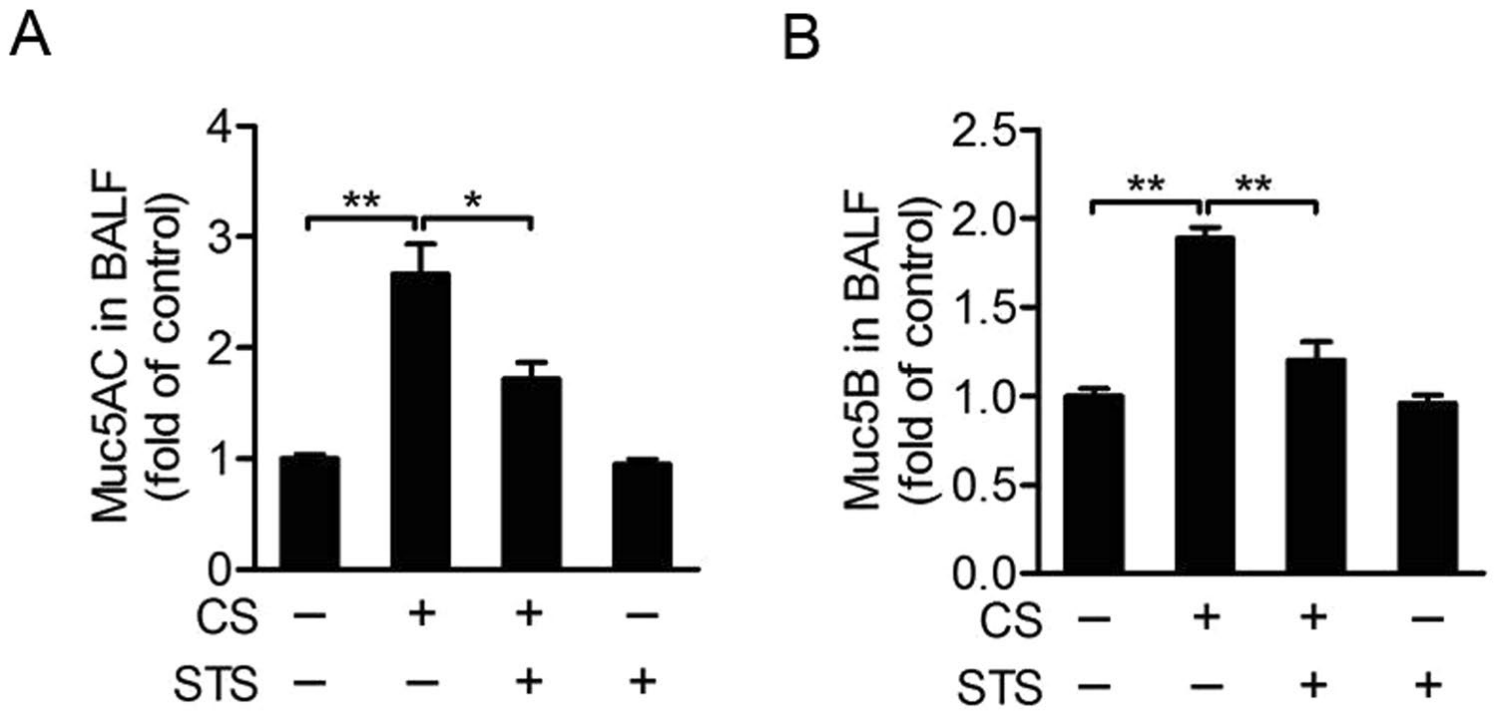
STS attenuates CS-mediated mucus hypersecretion in the airway. Comparison of levels of Muc5AC (**A**) and Muc5B (**B**) in BALF from CTL, CS, CS plus STS (CS + STS) and STS-treated mice. *P < 0.05and **P < 0.01 (n = 8–10 mice per group). STS, Sodium tanshinone IIA sulfonate; CS, cigarette smoke.

The expression of Muc5AC and Muc5B increased in bronchoalveolar lavage fluid (BALF), which was attenuated by STS inhalation (Muc5AC: CS group: 279.25 ± 28.70%, CS + STS group: 171.62 ± 16.60%; Muc5B: CS group: 188.68 ± 6.43%, CS + STS group: 135.27 ± 14.63%, percentage of the CTL group, Fig. 4). These results suggest that STS inhalation exhibits significant protective effect on COPD through lessening mucus hypersecretion in CS/LPS-induced COPD mice.

### STS inhibits CS-induced inflammation and oxidative stress in mouse lungs and in 16HBE cells

**Figure 5:**
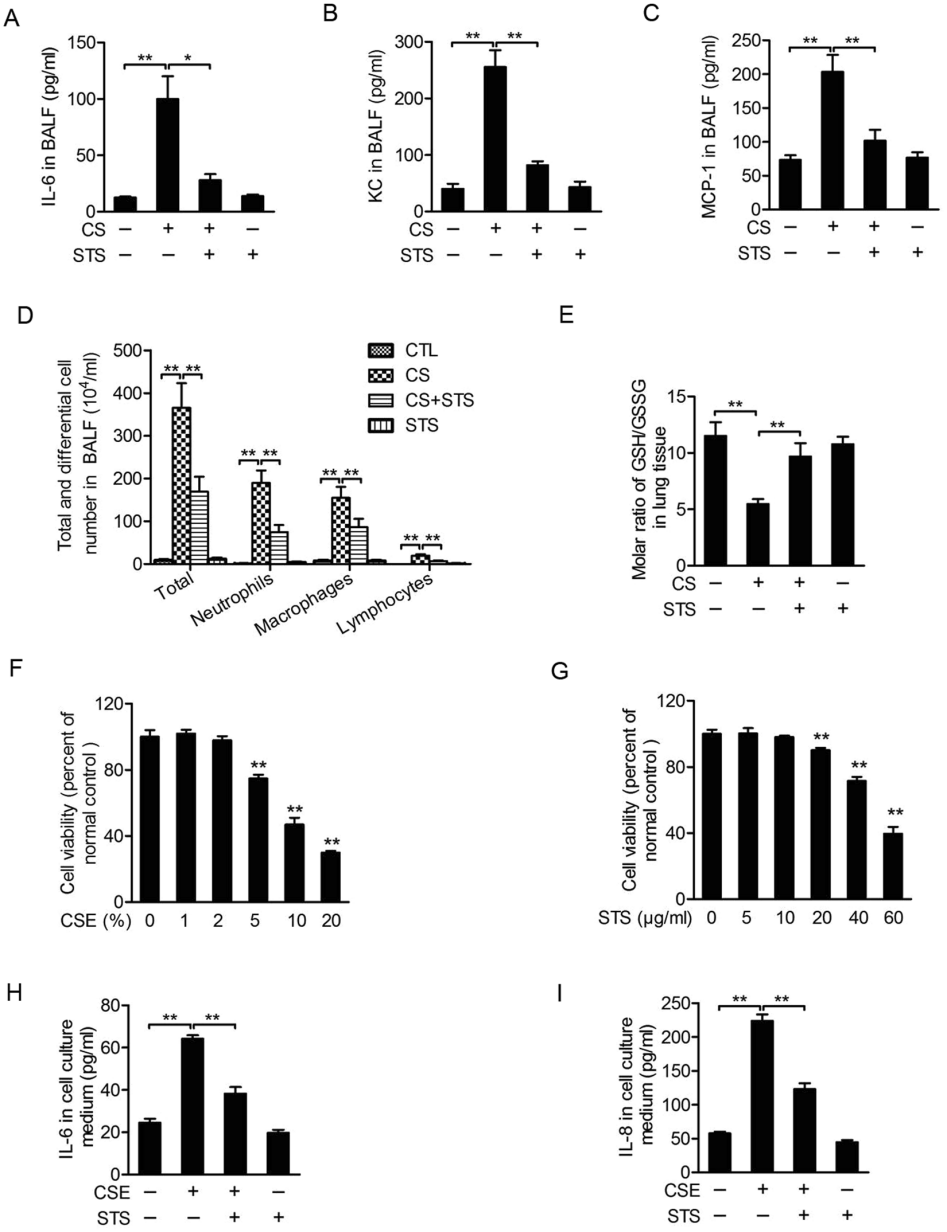
STS protects against lung inflammatory responses and oxidative stress in mouse COPD model. Comparison of levels of IL-6 (**A**), KC (**B**), MCP-1 (**C**) and cell counts (**D**) in BALF from CTL, CS, CS plus STS (CS + STS) and STS-treated mice. Comparison of GSH/GSSG ratio (**E**) in mouse lungs from CTL, CS, CS plus STS (CS + STS) and STS-treated mice. *P < 0.05 and **P < 0.01 (n = 8–10 mice per group). (**F–G**) Cells were treated with CSE (1%, 2%, 5%, 10%, 20%) or STS (5 μg/ml, 10 μg/ml, 20 μg/ml, 40 μg/ml and 60 μg/ml) for 48 hours and cell viability was measured by a CCK8 assay. 16HBE cells were treated with 2% CSE for 48 hours with or without 2 h of STS (10 μg/ml) pretreatment, the expression of IL-6 (**H**) and IL-8 (**I**) in cell culture medium was measured. **P < 0.01, n = 5. STS, Sodium tanshinone IIA sulfonate; CS, cigarette smoke; CSE, cigarette smoke extract; KC, keratinocyte chemoattractant; MCP-1, monocyte chemoattractant protein-1.

Based on anti-inflammatory functions of STS, we evaluated the effect of STS on lung inflammatory responses in COPD mouse model. As shown in Fig. 5, LPS and CS exposures caused mouse airway and lung inflammation, manifested by higher levels of IL-6 (Fig. 5A), KC (Fig. 5B) and MCP-1 (Fig. 5C) and increases in total and differential cell counts (Fig. 5D) in BALF. Inflammatory responses were attenuated by STS treatment (IL-6: CS group: 797.82 ± 161.61%, CS + STS group: 223.24 ± 42.8%; KC: CS group: 635.80 ± 74.90%, CS + STS group: 311.82 ± 75.58%; MCP-1: CS group: 277.19 ± 34.54%, CS + STS group: 138.46 ± 22.19%; Total cell: CS group: 4,244.18 ± 6.78%, CS + STS group: 1,966.57 ± 9.55%; Neutrophils: CS group: 24,802.36 ± 3895.37%, CS + STS group: 9,764.97 ± 2179.95%; Macrophages: CS group: 2,086.99 ± 348.08%, CS + STS group: 1,169.68 ± 255.08%; Lymphocytes: CS group: 6,665.51 ± 120.64%, CS + STS group: 2,374.56 ± 563.45%, percentage of the CTL group, Fig. 5A–D). We evaluated the oxidative stress by measuring the molar ratio of GSH/GSSG in lung tissue. STS attenuated the decline of GSH/GSSG ratio in lung tissue of CS-exposed mice (CS group: 47.52 ± 3.76%, CS + STS group: 83.97 ± 10.37%, percentage of the CTL group, Fig. 5E). We utilized human bronchial epithelial cells (16HBE) to determine whether STS also reduced CSE-induced inflammatory responses *in vitro*. The cell viability assay showed that high concentrated CSE (5%, 10% and 20%) and STS (20 μg/ml, 40 μg/ml and 60 μg/ml) induced obvious cytotoxicity indicated by substantial cell viability loss, whereas low concentrations of CSE (1% and 2%) and STS (5 μg/ml and 10 μg/ml) had no effects on cell viability (Fig. 5F–G). Therefore, we treated 16HBE with CSE (2%) to determine the effects of STS (10 μg/ml) on inflammatory mediator release. We found that STS (10 μg/ml) partially inhibited the effect of CSE (2%) on pro-inflammatory cytokine secretion in 16HBE cells (IL-6: CSE group: 261.98 ± 6.89%, CSE + STS group: 156.02 ± 12.99%; IL-8: CSE group: 388.96 ± 16.38%, CSE + STS group: 213.56 ± 15.23%, percentage of the CTL group, Fig. 5H–I). These data suggest that STS inhibits CS-induced inflammatory responses in mouse lungs and in 16HBE.

### STS reduces CS-induced activation of ERK/NF-κB pathway in mouse lungs and 16HBE cells

**Figure 6:**
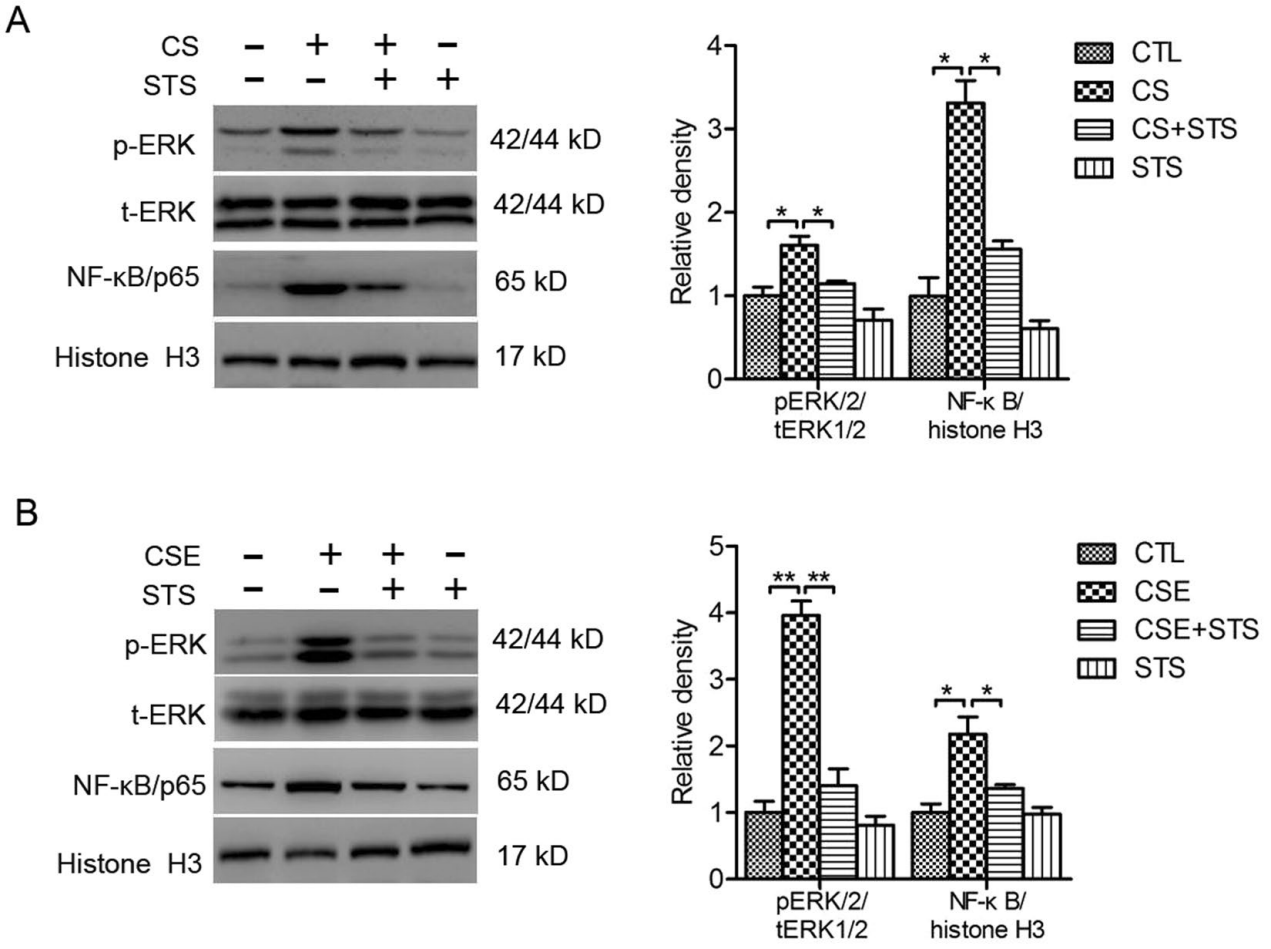
STS reduces CS-induced activation of ERK/NF-κB pathway in 16HBE cells and mouse lungs. (**A**) Levels of phosphorylated ERK1/2 in cytosol protein and levels of NF-κB in nuclear protein from lung tissue of mice exposed to CS with or without STS treatment. Full-length blots are presented in supplementary Figure 1A. *P < 0.05 and **P < 0.01, n = 3. CSE, cigarette smoke extract; CS, cigarette smoke. (**B**) 16HBE cells were treated with 2% CSE for 1 h with or without 2 h of STS pretreatment. Levels of ERK1/2 and phosphorylated ERK1/2 in cytosol protein and levels of NF-κB with histone H3 as loading control in nuclear protein. Full-length blots are presented in supplementary Figure 1B.

As shown in Fig. 6A, CS exposure induced phosphorylation of ERK1/2 (CS group: 396.11 ± 21.23%, percentage of the CTL group) without impacting cytosol ERK level. The nucleic distribution of NF-κB/p65 (CS group: 217.55 ± 25.81%, percentage of the CTL group) was also enhanced by CS exposure. STS administration attenuated the activation of ERK1/2 and NF-κB/p65 in lung tissue from CS-exposed mice (CS + STS group: pERK1/2: 160.47 ± 19.44%; CS + STS group: p65/NF-κB: 160.47 ± 19.44%, percentage of the CTL group, Fig. 6A). Simultaneously, CSE-induced activation of ERK1/2 (CSE group: 160.47 ± 10.99%, percentage of the CTL group) and NF-κB/p65 (CSE group: 331.22 ± 27.03%, percentage of the CTL group) in 16HBE cells was inhibited by STS treatment (CSE + STS group: pERK1/2: 114.63 ± 4.77%; p65/NF-κB: 155.73 ± 10.26%, percentage of the CTL group, Fig. 6B). These results indicate that STS reduces inflammatory responses through decreasing ERK1/2 and p65/NF-κB activation.

### STS protects against CS-induced reduction of CFTR in 16HBE cells and mouse lungs

**Figure 7:**
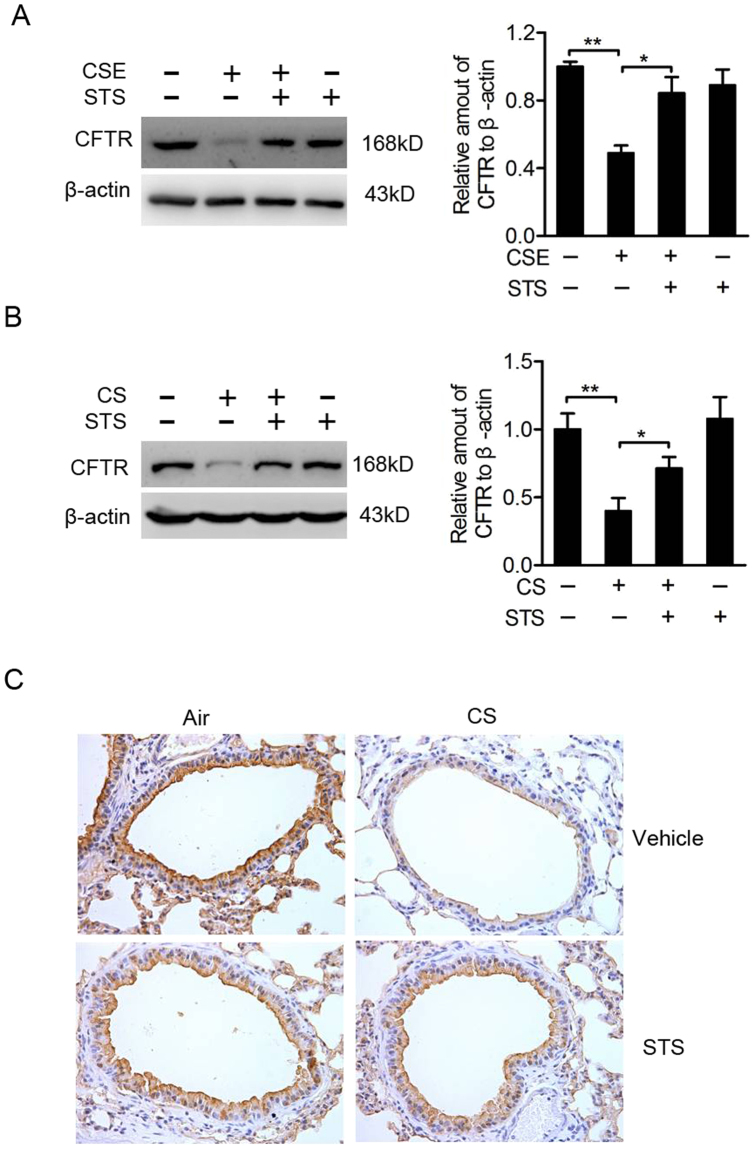
STS protects against CS-induced reduction of CFTR in 16HBE cells and mouse lungs. 16HBE cells were treated with 2% CSE for 48 h with 2 h of STS (10 μg/ml) pre-incubation. (**A**) The level of CFTR in total cell extract was determined, while β-actin used as internal control (n = 4 per group). Full-length blots are presented in supplementary Figure 2A. (**B**) Western blotting showed the impact of STS on CFTR expression in lungs from CTL, CS, CS plus STS (CS + STS) and STS-treated mice (n = 6 mice per group). Full-length blots are presented in supplementary Figure 2B. (**C**) Immunohistochemistry showed the impact of STS on CFTR expression in lungs from CTL, CS, CS plus STS (CS + STS) and STS-treated mice (n = 3 mice per group). *P < 0.05 and **P < 0.01. CFTR, cystic fibrosis transmembrane conductance regulator; CSE, cigarette smoke extract; CS, cigarette smoke; STS, Sodium tanshinone IIA sulfonate.

Sustained exposure to CS or CSE has been shown to reduce CFTR expression. As expected, CSE exposure reduced CFTR protein in 16HBE cells (Fig. 7A). STS treatment attenuated the reduction of CFTR protein caused by CSE incubation **(**CSE group: 48.93 ± 4.35%, CSE + STS group: 84.18 ± 9.55%, percentage of the CTL group, Fig. 7A). Similarly, STS administration attenuated the inhibitory effect of CS on CFTR protein abundance in mouse lungs, determined by Western blotting (CS group: 39.88 ± 9.85%, CS + STS group: 71.20 ± 8.53%, percentage of the CTL group, Fig. 7B) and immunohistochemistry (Fig. 7C). These results indicate STS protects against CS-induced reduction of CFTR level.

### CFTR inhibition abolishes the inhibitory effect of STS on CSE-induced IL-6 and IL-8 release, but increases CSE-induced activation in ERK/NF-κB pathway

**Figure 8:**
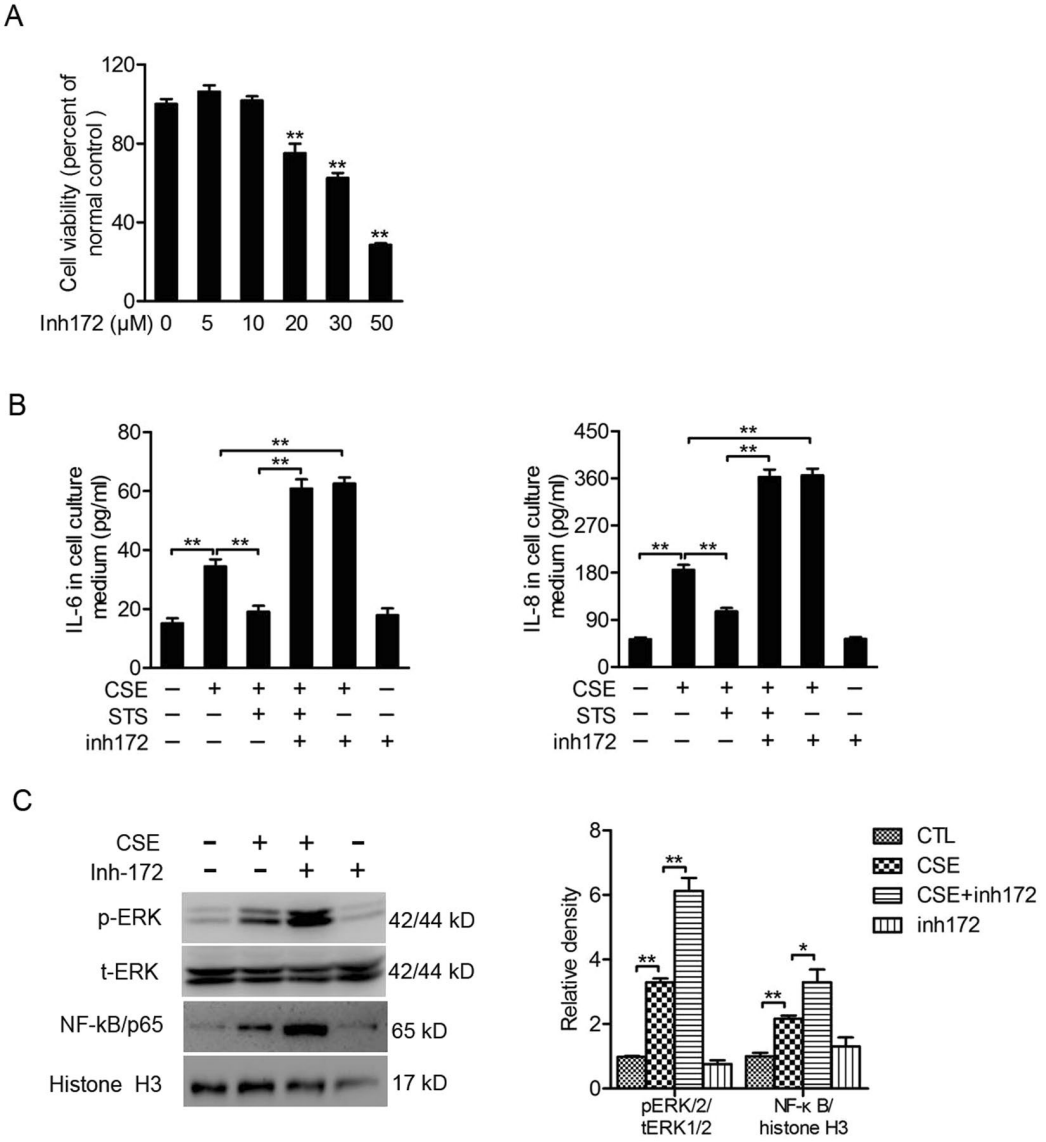
CFTR inhibition abolishes inhibitory effect of STS on CSE-induced release in IL-6 and IL-8, but increases CSE-induced activation in ERK/NF-κB pathway. 16HBE cells were treated with 2% CSE for 48 h with 2 h of STS (10 μg/ml) pre-incubation. In order to inhibit CFTR function, cells were treated with CFTR specific inhibitor CFTR-Inh172 (10 μM) 1 h prior to STS treatment. (**A**) cells were treated with CFTR-Inh172 (0–50 μM) for 48 hours and cell viability was measured by CCK8 assay. (**B**) IL-6 and IL-8 expression in cell culture medium was measured. (**C**) The levels of phosphorylated ERK1/2 in cytosol protein were determined with ERK1/2 as internal control (n = 3). The level of p65/NF-κB in nuclear extraction was determined with histone H3 as internal control (n = 6). Full-length blots are presented in supplementary Figure 3. *P < 0.05 and **P < 0.01, CFTR, cystic fibrosis transmembrane conductance regulator; CSE, cigarette smoke extract; CS, cigarette smoke; STS, Sodium tanshinone IIA sulfonate.

To determine the role of CS-induced inflammatory responses, 16HBE cells were treated with CSE (2%) for 48 h with or without STS (10 μg/ml) or CFTR-Inh172. Cell viability assay showed that high concentrated CFTR-Inh172 (20 μM, 30 μM and 50 μM) but not 10 μM induced obvious cytotoxicity (Fig. 8A). Thus, we used CFTR-Inh172 at 10 μM for following experiments. As shown in Fig. 8B, pretreatment with CFTR-Inh172 (10 μM, 48 h) significantly augmented CSE-induced IL-6 release (CSE group: 226.94 ± 15.65%, CSE + CFTR-Inh172 group: 401.37 ± 20.64%, percentage of the CTL group) and IL-8 (CSE group: 347.08 ± 17.57%, CSE + CFTR-Inh172 group: 679.34 ± 26.89%, percentage of the CTL group) in 16HBE cells. This was associated with an increase in phosphorylated ERK1/2and nuclear p65/NF-κB protein in 16HBE cells treated with CFTR-Inh172 (10 μM) compared to cells only exposed to CSE (pERK1/2: CSE group: 329.75 ± 10.85%, CSE + CFTR-Inh172 group: 612.91 ± 40.11%; p65/NF-κB: CSE group: 216.12 ± 9.92%, CSE + CFTR-Inh172 group: 329.43 ± 39.57%, percentage of the CTL group, Fig. 8C). Interestingly, protective role of STS on CSE-induced IL-6 and IL-8 release was abolished by CFTR-Inh172 (10 μM) treatment (Fig. 8B). Altogether, these results suggest that STS protects against CS-induced inflammatory responses in lung epithelial cells by maintaining CFTR levels.

## Discussion

Danshen and its derivative products including STS have been applied to treat cardiovascular and cerebrovascular diseases both in Asian and Western countries^9^. Our study provided evidence that a long-term aerosol inhalation of STS reduced lung physiological and pathological manifestations in mice exposed to CS. This was associated with the reduction of CS-induced oxidative stress and inflammatory responses *in vivo* in mice and *in vitro* in cells. This is corroborated by another finding showing that STS is effective in clinical treatment of pulmonary hypertension, a common complication of COPD^13^.

LPS helps to induce emphysema in shorter time than pure CS exposure (couples of months vs 6–8 months)^14^. We used both LPS and CS exposures in mice, and treated them with STS. STS was described as an antioxidant to reduce oxidative stress and inflammatory responses, which is associated with its function such as scavenging oxygen free radicals, preventing lipid peroxidation^15^, inhibiting low density lipoprotein oxidation^16^, increasing Zn superoxide dismutase (SOD) activity as well as mRNA and protein expression^17^, activating the Nrf2 pathway^18^ and inhibiting NF-κB and MAPK signaling pathways^19^. In the present study, we found that STS treatment significantly reduced lung inflammatory responses to CS exposure. This may be due to inhibition of NF-κB and MAPK signaling pathway, which is shown in our and previous studies^19^. A broad spectrum of anti-inflammatory drugs, including inhibitors of the pro-inflammatory enzymes PDE4, Janus kinases, NF-κB kinase, p38 mitogen-activated protein kinase, and PI3 kinase-γ and -δ, were developed for COPD treatment, but side effects after oral administration limited their clinical application^20^. Unlike significant advances in the development of long-acting bronchodilators, it has been proved difficult to find safe and effective anti-inflammatory treatments for COPD^20^. As a medicine used in traditional Chinese medicine, side effects and safety of Danshen and Danshen products have been fully evaluated in clinical practice^9^. Indeed, we found that increases of organ and body indices were attenuated by STS treatment, suggesting the dose of STS (5 mg/kg, 30 min per session, twice per day) here to treat COPD did not cause a detectable toxic effect. Based on our findings, STS is a potential drug for preventing COPD, which could be conveniently delivered by aerosol inhalation.

Cystic fibrosis transmembrane conductance regulator (CFTR), an epithelial anion channel, is expressed mainly in exocrine tissues. In lung, loss of CFTR function results in mucociliary clearance defect, airway surface liquid depletion, chronic bacterial infection and airway chronic inflammation^21^. CS-mediated CFTR dysfunction results in inflammation and oxidative stress, and both are involved in the pathogenesis of COPD emphysema^22^. Therefore, pharmacological approaches aimed at enhancing CFTR expression and/or function could be potentially beneficial to COPD.

In response to CS exposure, level of CFTR was reduced in mouse lungs and in lung epithelial cells. Recent studies showed CFTR gene expression in human airway epithelial cells was regulated by oxidative stress^23,24^. Current findings and others showed that STS protected against oxidative stress^25^. These findings suggest that STS protects against CFTR reduction through reducing oxidative stress. GSH, one of the most important antioxidants against free radicals in lungs, affords protection against oxidative damage on the alveolar epithelial fluid interface^26^. Compared with mice without STS treatment, higher ratio of free GSH to GSSH was found in lung tissue from COPD model mice treated with STS, which means a better cellular stability against oxidative damage^27^. These findings suggest that there is an interaction between CFTR and oxidative stress, which is disrupted by CS exposure.

Mucus hypersecretion, an important factor of predicting the morbidity and mortality in COPD, is contributed by CS-induced acquired CFTR dysfunction, while it can be markedly improved by the CFTR potentiator *in vitro* ^28^. Our preliminary studies have shown that STS inhalation down-regulates Muc5AC and Muc5B levels in BALF of mice with COPD. This was in agreement with the finding that STS inhalation reduced CS-induced goblet cell hypertrophy and hyperplasia of the airway epithelium in COPD mice. The pathogenesis of airway mucus hypersecretion induced by CS, which is complicated and incompletely understood, may involve inflammation, oxidative stress, signal transduction and decreased expression of CFTR^29^. Treatments should counteract each factor contributing to airway mucus hypersecretion. Our study shows that treatment with aerosolized STS twice daily (5 mg/kg) is associated with significant improvements in mucus clearance. The mechanism of benefit is thought to be attributed to inflammation and oxidative stress inhibition, as well as CFTR expression maintenance.

MAPK signaling pathways regulate pro-inflammatory mediator IL-8 expression in cystic fibrosis lung epithelial cell lines^30^. Inhibition of CFTR function enhanced inflammatory responses to CS by activation of p38/ERK MAPK or NF-κB pathway in airway bronchial epithelial cells^31,32^. This is consistent with our findings that pharmacological inhibition of CFTR augments CS-induced IL-6 and IL-8 expression as well as MAPK and NF-κB activation, despite CFTR inhibition by CFTR-Inh172 (10 µg/ml) alone did not cause inflammatory responses in lung epithelial cells. The mechanisms underlying the discrepancies between these findings are not clear. Interestingly, protective role of STS on CS-induced inflammatory responses was abolished in response to CFTR inhibition. All these findings suggest that STS reduces CS-induced inflammatory responses by attenuating CFTR reduction, although there is a need to use CFTR knockout cells to further unravel its role in CS-induced inflammatory and injurious responses.

In summary, our current study demonstrates that STS inhalation effectively attenuates CS-induced clinical or pathological presentations of COPD, such as emphysema, collagen deposition in the small airway, goblet cell hyperplasia of airway epithelium and lung function decline. This was associated with protective effects of STS on CS-induced oxidative stress, NF-κB-dependent inflammatory responses. Furthermore, CS-induced down-regulation of CFTR is also prevented by STS. STS could be a potential anti-inflammatory drug for COPD.

Nevertheless, our study has some limitations, such as not figuring out the specific molecular mechanism underlying that STS inhibited CS-induced CFTR reduction in COPD. However, the study has already provided evidence to support that STS inhalation has clinical efficiency, feasibility and relatively lower cost compared with classic COPD medication as a potential drug for COPD. STS inhalation is a valuable candidate worthy of further investigation for future CS-induced COPD therapy.

## Methods

### Animal model

Wide-type C57BL/6 J male mice (6–8 weeks old) were purchased from the Nanjing BioMedical Research Institute of Nanjing University (Nanjing, China). Animals were housed in the specific pathogen-free facilities and the Animal Care and Use Committee of Guangzhou Medical University approved all experimental protocols. All methods were performed in accordance with the guidelines and regulations approved by the Animal Care and Use Committee of Guangzhou Medical University. The cigarettes used in the mouse model establishment were Plum brand filtered cigarettes (Guangdong Tobacco Industry, China) and each cigarette yields 11 mg tar, 1.0 mg nicotine and 13 mg carbon monoxide. A COPD model was established using CS exposure plus intranasal inhalation of Lipopolysaccharide (LPS) **(**Fig. 1A). Briefly, mice received LPS (7.5 μg/mouse in 50 μl saline; L8643, Sigma-Aldrich) or saline (vehicle control) by intranasal inhalation at the 1^st^ and 14^th^ day. Meanwhile, mice were exposed to CS (9 cigarettes/h, 2 hours/session, twice/day and 6 days/week in a whole-body exposure chamber) from day 0 to 60 except for the days giving LPS. For STS (Jiangsu Carefree Pharmaceutical, Nanjing, China) treatment, mice were given saline or STS (5 mg/kg, 30 min per session, twice per day) by airway inhalation with a PARI Nebuliser in a whole-body exposure chamber for 30 min daily before being exposed to CS. Finally, all mice were subjected to lung function analysis, and sacrificed at day 61.

### Lung function measurement

Lung function was evaluated using the Forced Pulmonary Maneuver System (Buxco Research Systems, Wilmington, North Carolina, USA) according to the manufacturer’s protocol. Briefly, mice anesthetized with pentobarbital (50 mg/kg body weight) were tracheostomized, intubated and put in the body chamber of the system. The average breathing frequency of anesthetized animals was forcibly set at 150 breaths/min. Three semiautomatic maneuvers were carried out: Boyle’s law functional residual capacity (FRC), quasi-static pressure volume (PV) and fast flow volume (FV) maneuver. The FRC was determined with Boyle’s law FRC. To measure total lung capacity (TLC) and chord compliance (Cchord), the quasi-static PV maneuver was performed. With the fast FV maneuver, forced expiration volume in 50 milliseconds (FEV50) and forced vital capacity (FVC) were measured.

### Hematocrit measurement

Briefly, at the terminal of chronic CS exposure, whole blood was collected into capillary tubes (0.5 mm outside diameter, VWR Scientific, Radnor, PA) via right ventricle puncture with K_2_EDTA as an anticoagulant and centrifuged at 7,000 rpm for 5 min, and read on a hematocrit chart (VWR Scientific).

### BALF analysis

The left lung was injected with three sequential 0.6 ml of ice cold saline intra-tracheally and slowly. A total of 1 ml BALF was collected and centrifuged at 500 g for 10 minutes at 4 °C to pellet the cells. The cell pellets were re-suspended in 1 ml saline for total cell counts using haemacytomer. The cells were subjected to Giemsa staining for differential counting of neutrophils, macrophages and lymphocytes. The supernatant was stored at −80 °C for pro-inflammatory cytokine detection.

### Histopathology and immunohistochemistry

The left lung specimens of mice were fixed in 10% neutral buffered formalin for 24 h, embedded in paraffin wax, and cut into 4 mm thick slices stained with hematoxylin and eosin (H&E) to evaluate morphological changes. The Image Analysis Software Image-Pro Plus 6.0 was used to assess the average alveolar intercept, which means the ratio of total alveolar length to the number of alveoli per field under microscopy. For immunohistochemistry, the slices were immunostained with anti-CFTR antibody (1:100 ACL006AN0602, Alomone Labs, Jerusalem, Israel) and developed by diaminobenzidine (DAB) solution according to manufacturer’s instructions. Periodic Acid-Schiff (PAS) stain (Shanghai Sun Biotechnology, Shanghai, China) was utilized to detect goblet cells in the airway epithelium following manufacturer’s instructions. Masson Trichrome stain (KGMST-8003) was utilized to detect collagen deposition of small airway according to manufacturer’s instructions. Semi-quantitative analyses of positive area in lungs were completed by two independent investigators using the Image-Pro Plus 6.0. At least three fields per mouse were taken.

### Measurement of liver, spleen, and kidney indices

The liver, spleen, or kidneys were weighed and each was calibrated with whole body weight.

### Preparation of nuclear protein extract

Lung tissue or 16HBE cells were extracted with NE-PER nuclear and cytoplasmic reagents (Thermo Scientific) containing protease inhibitor cocktail according to the manufacturer’s instructions. Histone H3 served as the loading control in Western blotting of nuclear proteins.

### Preparation of CSE

CSE was freshly prepared from Plum brand filtered cigarettes within 30 min prior to treatments according to a previously described protocol^33^. The acquired CSE suspension in yellowish color with optical density (OD) at 405 nm 0.506 ± 0.008 was adjusted to pH7.4, passed through a 0.22 μm filter to remove bacteria and particles and considered as concentration 100% in cell treatments.

### Cell viability assay

The cell viability of 16HBE cells was determined by using a CCK8 assay kit (Sigma-Aldrich) according to the manufacturer’s instruction. Briefly, the cells (5,000 cells/well) were seeded in 96-well plates, cultured overnight, treated with CSE (0–20% in medium containing 1% FBS) or STS (0–60 μg/ml) or CFTR-Inh172 (0–50 μM) for 48 h. Subsequently, CCK8 solution (10 µl) was added to each well and incubated for 2 h. Finally, the absorbance at 450 nm was read in a microplate absorbance reader (Thermo Scientific, Waltham, MA). Cell viability was presented as percentage of normal control cells, i.e. cells without CSE or STS or CFTR-Inh172 treatment.

### Western blotting

Mouse lung tissue or cells were homogenized by RIPA lysis buffer with 1% protease inhibitor cocktail (Sigma-Aldrich) to extract the total protein or were separated to extract the nuclear fractions using a NE-PER nuclear and cytoplasmic extraction reagents (Thermo scientific). The same amount of protein was separated by SDS-PAGE (Bio-Rad, Hercules, CA) and blotted with primary antibodies (anti-mouse CFTR antibody, 1:1000, ACL006AN0602, Alomone Labs, Jerusalem, Israel; anti-human CFTR antibody, 1:1000, #2269; anti-Erk1 (pT202/pY204) + Erk2 (pT185/pY187) antibody, 1:1000, ab76299; anti-ERK1/2 antibody, 1:1000, #4695; anti-p65/NF-κB antibody, 1:1000, #6956S; anti-histone H3 antibody, 1:1000, #9715; and anti-β-actin antibody,1:4000, sc-47778). Anti-phosphorylated ERK1/2 antibody was from Abcam (Cambridge, UK). Antibodies against human CFTR, ERK1/2, NF-κB/p65 and histone H3 were purchased from Cell Signaling Technology (Beverly, MA, USA). Anti β-actin antibody was obtained from Santa Cruz Biotechnology (Dallas, TX, USA). The peroxidase-conjugated secondary antibody was from Sigma-Aldrich (St Louis, MO, USA). The signal of bound antibodies was detected using the Immun-Star HRP Chemiluminescent kit (Bio-Rad, Hercules, CA).Western blot image was obtained by Tanon 5200 chemiluminescence imaging system (Shanghai Tanon Science & Technology, Shanghai, China). Semi-quantitative analyses of immunoblots were obtained by using the Image J.

### Cell culture

Human bronchial epithelial cells 16HBE were purchased from Cell Bank of the Chinese Academy of Sciences (Shanghai, China) and cultured with DMEM medium added with 10% FBS, 100 ug/ml penicillin and 100 μg/ml streptomycin in a humidified incubator at 37 °C with 95% (v/v) air and 5% (v/v) CO_2_. The cells were treated with CSE (2%) and STS (10 μg/ml) with or without CFTR inhibitor CFTR-Inh172 (10 μM, Sigma) in serum starved medium.

### Enzyme-linked immunosorbent assay (ELISA)

The levels of interleukin (IL)-6, keratinocyte chemoattractant (KC), monocyte chemoattractant protein-1 (MCP-1) and IL-8 in BALF or in cell culture medium were measured by ELISA. Briefly, human or mouse IL-6 (88–7066/88–7064), mouse MCP-1 (88–7391) and human IL-8 (88–8086) were measured using the ELISA kits obtained from eBioscience (San Diego, CA) following the manufacturer’s protocols. KC in BALF was detected using the capture and detection antibodies obtained from R&D Systems (Minneapolis, MN) according to protocol described previously^34^. The capture antibodies for Muc5AC (sc-21701) and Muc5B (sc-135508) were obtained from Santa Cruz Biotechnology (Santa Cruz, CA) and the corresponding detection antibodies were obtained from KPL (Gaithersburg, MD). ELISA signals were observed using the TMB Substrate Reagent Set (BD Biosciences Pharmingen, San Diego, CA).

### Glutathione fluorometric assay

The ratio of GSH/GSSG was measured in lung using the GSH/GSSG ratio detection assay kit (BioVision, Milpitas, CA) according to manufacturer’s instruction. Briefly, lung tissue was homogenized using cold glutathione assay buffer. To every 60 μl of homogenate was added 20 μl of prechilled perchloric acid (PCA, 6 N). The solution was mixed, centrifuged and the supernatant was kept at −80 °C. The PCA-preserved samples were assayed for glutathione after addition of ice cold KOH (6 N, 20 μl to 40 μl per sample) to precipitate PCA and centrifuged. For GSH measurement, 10 μl of sample was added to 90 μl of assay buffer. For GSSH measurement, 10 μl of sample was added to 70 μl of assay buffer, followed by addition of a quencher buffer to quench GSH and a reducing agent to destroy excessive GSH quencher and transform GSSG to GSH. Finally, o-phthalaldehyde (OPA) probe was added to each sample and the relative fluorescence units (RFU) were read in a fluorescence plate reader with λ_ex_ = 340 nm and λ_ex_ = 420 nm. A standard curve with known concentrations of GSH supplied with the kit was used to calculate GSH concentration. The results were expressed as molar ratio of GSH/GSSH.

### Statistical analysis

Data were analyzed with ANOVA or two-tailed Student’s t-test. If significant F-ratios were acquired with ANOVA, pair-wise comparison of mean was carried out with Boferroni analysis. Data are presented as mean ± SEM. “n” means the number of repeats in cell experiments or the number of animals. P < 0.05 and P < 0.01 were considered significant.

## Electronic supplementary material

Supplementary information
