# Supplementary material for: Tanshinone IIA sulfonate protects against cigarette smoke-induced COPD and down-regulation of CFTR in mice
Source: Sci Rep. 2018 Jan 10;8:376. doi: 10.1038/s41598-017-18745-5 (PMC5762644; doi:10.1038/s41598-017-18745-5)
Supplement: Supplementary file 1 — Supplementary information [file 41598_2017_18745_MOESM1_ESM.pdf]

Supplementary information

Tanshinone IIA sulfonate protects against cigarette smoke-induced COPD and down-regulation of CFTR in mice

Defu Li[1]*, Jian Wang[1]*, Dejun Sun[4]*, Xuefang Gong[1], Hua Jiang[1], Jiaze Shu[1], Ziyi Wang[1], Zhen Long[1], Yiguan Chen[5], Zili Zhang[1], Liang Yuan[1], Ruijuan Guan[1], Xue Liang[1], Ziying Li[1], Hongwei Yao[1,6], Nanshan Zhong[1], Wenju Lu[1, 2, 3 §]

[1]State Key Laboratory of Respiratory Diseases, Guangzhou Institute of Respiratory Disease, The First Affiliated Hospital, Guangzhou Medical University, Guangzhou, Guangdong, China; [2]Department of Laboratory Medicine, The First Affiliated Hospital, Guangzhou Medical University, Guangzhou, Guangdong, China; [3]Sino-French Hoffmann Immunology Institute, Guangzhou Medical University, Guangzhou, Guangdong, China; [4]Department of Respiratory Medicine, The People's Hospital of Inner Mogolia; [5]Faculty of Biology, Medicine and Health, University of Manchester, Manchester City, Britain; [6]Department of Molecular Biology, Cell Biology & Biochemistry, Brown University Warren Alpert Medical School, Providence, RI, USA.

Running title: TIIA inhibits cigarette smoke-induced COPD

*These authors contributed equally to this article.

§**Correspondence**:
Wenju Lu, MD, PhD
Professor of Respiratory Medicine
State Key Lab of Respiratory Diseases
The First Hospital of Guangzhou Medical University
Rm501E, Building 16
195 Dongfengxi Road, Guangzhou, China
Email: wlu92@yahoo.com
Tel: 86-20-83205040; Fax: 86-20-83205162
Email: wlu92@yahoo.com

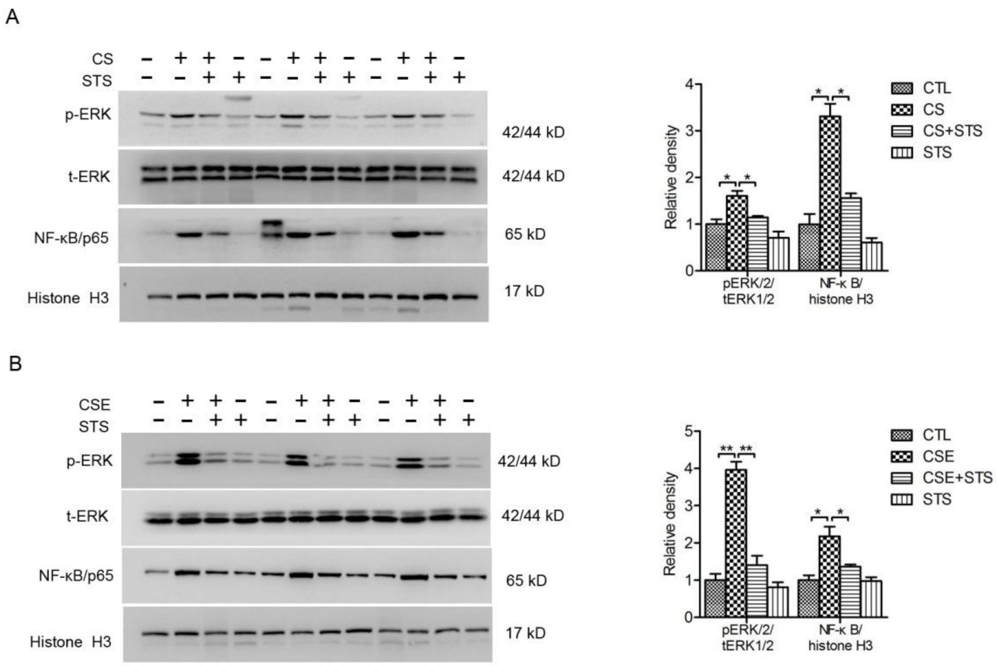

Supplementary figure 1. STS reduces CS-induced activation of ERK/NF-κB pathway in 16HBE cells and mouse lungs. (A) Levels of phosphorylated ERK1/2 in cytosol protein and levels of NF-κB in nuclear protein from lung tissue of mice exposed to CS with or without STS treatment**.** *P< 0.05 and **P< 0.01, n=3. CSE, cigarette smoke extract; CS, cigarette smoke. (B) 16HBE cells were treated with 2% CSE for 1 h with or without 2 h of STS pretreatment. Levels of ERK1/2 and phosphorylated ERK1/2 in cytosol protein and levels of NF-κB with histone H3 as loading control in nuclear protein**.** *P< 0.05 and **P< 0.01. CFTR, cystic fibrosis transmembrane conductance regulator; CSE, cigarette smoke extract; CS, cigarette smoke; STS, Sodium tanshinone IIA sulfonate.

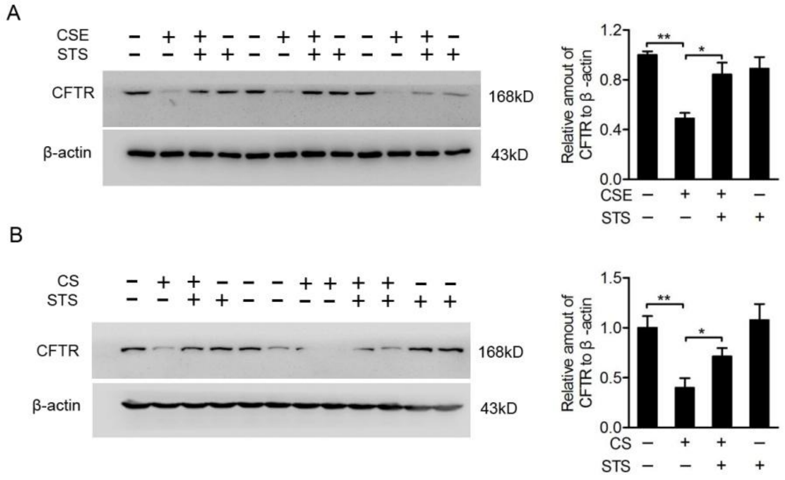

Supplementary figure 2. STS protects against CS-induced reduction of CFTR in 16HBE cells and mouse lungs. 16HBE cells were treated with 2 % CSE for 48 h with 2 h of STS (10 μg/ml) pre-incubation. (A) The level of CFTR in total cell extract was determined with β-actin as internal control (n=4 per group). (B) Western blotting tested the impact of STS on CFTR expression in lungs from CTL, CS, CS plus STS (CS+STS) and STS-treated mice (n=6 mice per group). *$P < 0.05$ and **$P < 0.01$. CFTR, cystic fibrosis transmembrane conductance regulator; CSE, cigarette smoke extract; CS, cigarette smoke; STS, Sodium tanshinone IIA sulfonate.

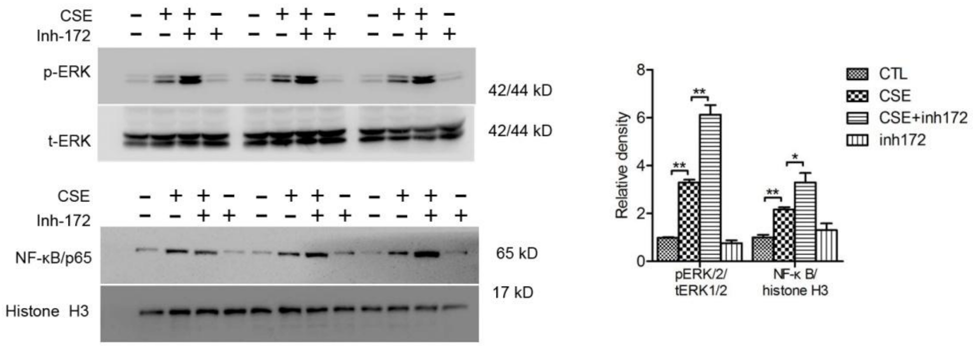

Supplementary figure 3. CFTR inhibition increases CSE-induced activation in ERK/NF-κB pathway. 16HBE cells were treated with 2% CSE for 1 h with 2 h of STS (10 μg/ml) pre-incubation. In order to inhibit CFTR function, cells were treated with CFTR specific inhibitor CFTR-Inh172 (10 μM) 1 h prior to STS treatment. The levels of phosphorylated ERK1/2 in cytosol protein were determined with ERK1/2 as internal control (n=3). The level of p65/NF-κB in nuclear extraction was determined with histone H3 as internal control (n=6). *P< 0.05 and **P< 0.01, CFTR, cystic fibrosis transmembrane conductance regulator; CSE, cigarette smoke extract; STS, Sodium tanshinone IIA sulfonate.
